# Supplementary material for: Navigating a New Normal: A Qualitative Look at Long-Term Care Planning for Children with Intellectual Disabilities Post-COVID-19
Source: Healthcare (Basel). 2024 Dec 11;12(24):2512. doi: 10.3390/healthcare12242512 (PMC11728057; doi:10.3390/healthcare12242512)
Supplement: Supplementary file 1 [file healthcare-12-02512-s001.zip › healthcare-3314607-supplementary.pdf]

Supplementary Table S1. Interview guide.

No.	Probing questions
1.	Please describe the family member with an ID you provide care for.
2.	What situations or events make family caregivers of persons with ID start thinking about the need for long-term care planning?
3.	As a carer for your family member with ID, what problems have you faced in trying to think or make plans for their long-term care when you cannot do it anymore?
4.	Can you describe your experiences as a family caregiver in discussing future long-term care with others and in making those arrangements for your relative with ID?
5.	In what ways has the COVID-19 pandemic affected or influenced long-term care planning for your family member with ID?
